# An unusual case report of primary vitreoretinal lymphoma

**DOI:** 10.1186/s12886-018-0860-9

**Published:** 2018-09-14

**Authors:** Shi Zhuan Tan, Laura R. Steeples, Ramandeep Chhabra, Nicholas P. Jones

**Affiliations:** 1 Centre for Ophthalmology and Vision Sciences, Faculty of Medical and Human Sciences, Medicine and Health, University of Manchester, Manchester, UK; 2 Centre for Biostatistics, Division of Population Health, Health Services Research and Primary Care, School of Health Sciences, Faculty of Biology, Medicine and Health, University of Manchester, Manchester, UK; 3 Manchester Academic Health Science Centre, Manchester Royal Eye Hospital, Central Manchester University Hospitals NHS Foundation Trust, Manchester, UK

**Keywords:** Vitreoretinal lymphoma, Central nervous system lymphoma

## Abstract

**Background:**

Primary vitreoretinal lymphoma (PVRL) is a rare ocular condition and its diagnosis remains a challenge. The clinical presentation is variable and it can masquerade as chronic intermediate or posterior uveitis. We report an unusual case of primary central nervous system lymphoma (PCNSL) presenting as migrating retinal lesions with unique shapes. The diagnostic challenges are described and the clinical features of intraocular lymphoma are reviewed.

**Case presentation:**

A 53 year-old gentleman presented with unilateral visual disturbance and a wide area of retinal whitening with sharp borders temporal to the macula, corresponding to hyper-reflective subretinal changes on optical coherence tomography (OCT). The lesion resolved spontaneously after 6 weeks but was replaced by multiple punctate sub-retinal and sub-retinal pigment epithelial lesions. The second eye was involved 4 months later and there were new areas of hyper-reflective changes in both eyes, which migrated nasally within a week, with no evidence of scarring in the previous sites. The diagnosis of diffuse B-cell lymphoma was made on brain biopsy when the patient subsequently presented with acute confusion and magnetic resonance imaging brain scan showed a frontal lesion. Following systemic chemotherapy, the sub-retinal changes resolved with complete restoration of retinal architecture and improvement in visual acuity.

**Conclusion:**

We report an unusual case of PVRL presenting as migrating retinal lesions with unique shapes. PVRL is a great imitator and a high index of clinical suspicion is required in unexplained ocular lesions to prevent a delay in diagnosis.

## Background

Primary central nervous system lymphoma (PCNSL) is a rare malignancy that is confined to the brain, spinal cord, eyes, and leptomeninges. Most cases are diffuse large cell non-Hodgkin B-cell lymphoma [1]. It represents only 2–4% of intracranial neoplasms and 4–6% of extranodal lymphomas [2]. Most cases occur sporadically but a compromised immune system has been found to be a predisposing factor [3, 4]. Intraocular lymphoma is a subset of PCNSL. Most intraocular lymphomas are primary vitreoretinal lymphoma (PVRL) that involves the retinal pigment epithelium and vitreous, as opposed to secondary metastatic systemic lymphoma that usually involves the uvea [5, 6].

Diagnosis of PVRL remains a challenge as it can mimic chronic posterior uveitis. Kimura et al. [7] and Fardeau et al. [8] reported the clinical manifestation of PVRL in their large case series. The commonest symptoms identified in these studies were floaters and painless moderate loss of vision and the commonest clinical signs reported included moderate vitritis, RPE pigmentary changes and sub-retinal infiltrate, which was found in 50% of the cases [7, 8]. The most useful signs for distinguishing lymphoma from other causes of uveitis in the French study were better vision, less anterior chamber flare, fewer cases with posterior synechiae, and a lack of optic disc swelling, epiretinal membrane, or retinal vasculitis [8].

We report a case of PVRL presenting with very unsual and migrating retinal lesions in the absence of vitritis.

## Case presentation

A 53- year old gentleman presented to the emergency eye service with a 1 week history of right visual disturbance with constant multi-coloured photopsia and reduced visual acuity. There was no significant past ocular or medical history. He was involved in a road traffic accident (RTA) 3 weeks earlier when his car was hit from the back but he sustained no injuries at that time. He was otherwise systemically well.

**Fig. 1:**
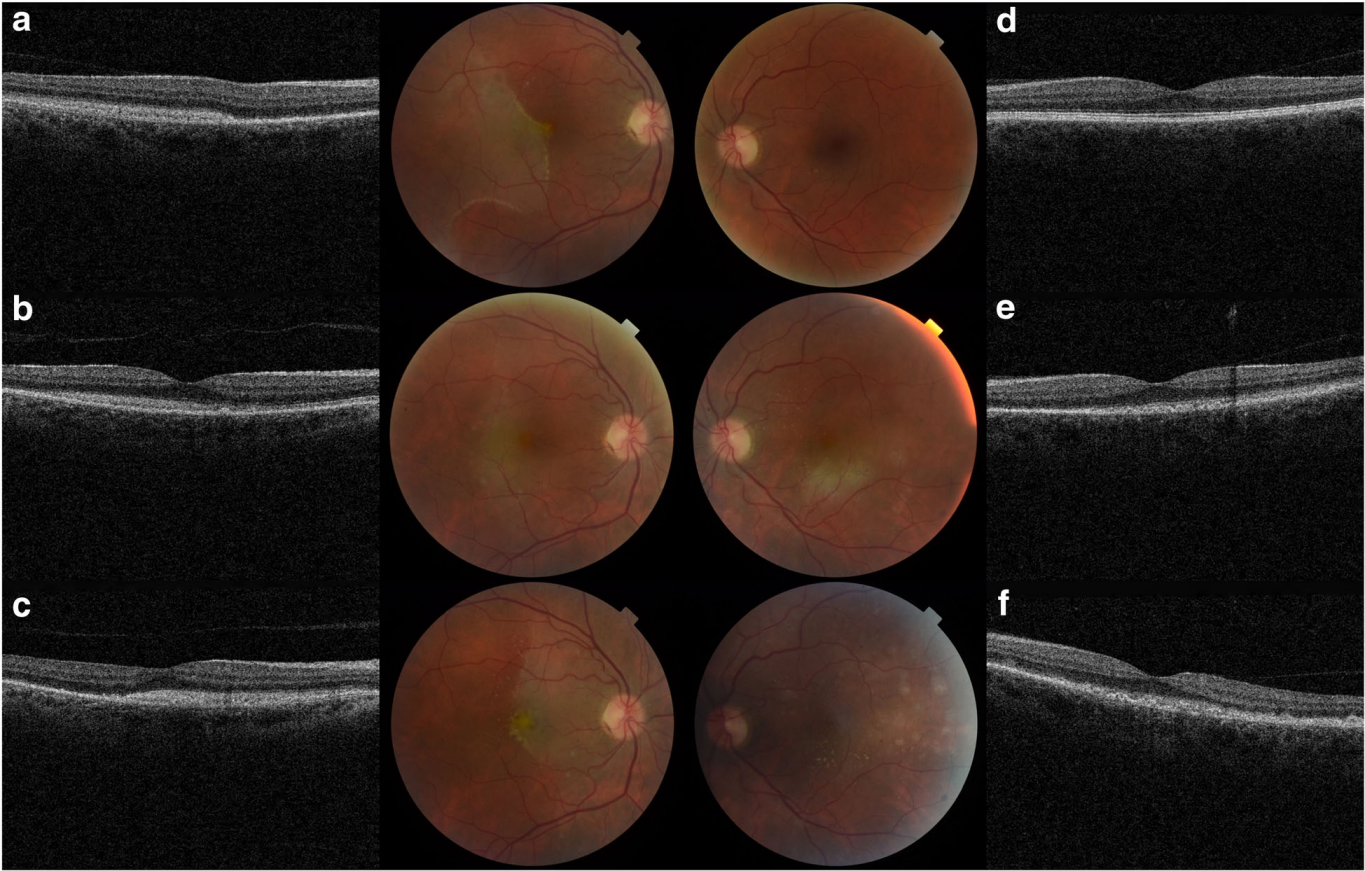
Colour fundus photography and Spectral Domain OCT (Topcon Medical Systems Inc., Newbury, UK) images. **a** Right fundus image showing a large area of retinal whitening with well-demarcated borders at first presentation and yellow spots superior to fovea. Hyperreflective subretinal changes was noted on OCT scan and disruption of the ellipsoid zone (EZ) nasal to the fovea was also noted. **b** Evolution of the right retinal lesion with corresponding hyper-reflectivity of the EZ on OCT at second presentation (4 months later). **c** A week after the second presentation, the area of retinal whitening was found to have migrated nasally. **d** Left normal fundus and OCT at presentation. **e** Left fundus showing an area of retina whitening with similar hyperreflective subretinal changes at second presentation. **f**. Left fundus showing spontaneous resolution of the retina whitening 1 week after second presentation but numerous yellow deposits at the subretinal and sub-RPE layers were evident

The best-corrected visual acuity (BCVA) was 6/12 in the right and 6/6 in the left eye. There were no anterior chamber or vitreous cells and no vitreous haze. In the right eye a large white deep retinal lesion, with an opaque appearance, was identified in the temporal retina, extending into the macular area and transecting the fovea (Fig. 1a). The lesion had a unique shape and margins: the nasal aspects of the lesion had a geometric like shape, with defined linear straight and curved edges, and a pronounced bright white border; the remaining border was less defined and homogenous with the lesion. Spectral domain optical coherence tomography (SD-OCT, Topcon Medical Systems Inc., Newbury, UK) within the lesion showed hyper-reflective sub-retinal change (Fig. 1a). Beyond the observed lesion edge, the ellipsoid zone (EZ) was disrupted in the nasal macular (Fig. 1a) Small, well-defined creamy white sub-retinal lesions were also evident in the superior macular area of the right eye (Fig. 1a). The left eye was unremarkable. (Fig. 1d).

The history of RTA led to consideration of commotio retinae, secondary to presumed whiplash injury from the car accident. Investigations for inflammatory and infective pathologies were negative including normal inflammatory markers, autoimmune screen, serum angiotensin enzyme, chest x-ray and syphilis testing.

Six weeks later, the patient reported full resolution of symptoms, including photopsia but the right BCVA remained the same. The area of retinal whitening and corresponding hyper-reflective sub-retinal OCT changes had resolved. Partial restoration of the EZ in the nasal macular was noted compared to earlier OCT. Multiple small macular sub-retinal pigment epithelium (RPE) deposits had appeared, with corresponding hyper-autofluorescence on fundus autofluorescence (FAF). The left eye remained normal.

The patient was lost to follow-up and re-presented 4 months later with recent onset blurred vision. The BCVA had deteriorated to 6/15 right and 6/19 left. There was no cellular activity in the vitreous but the posterior vitreous face appeared condensed and thickened. New retinal lesions were evident in the maculae of both eyes: in the right, a long, vertical lesion with ill-defined edges and different orientation, shape and margin appearance to the original lesion, was present; (Fig. 1b) in the left, a similar, ill-defined white retinal lesion inferior to the fovea was noted (Figure 1e). OCT within these lesions demonstrated hyper-reflective sub-retinal change, similar to the original lesion (Fig. 1b and e). More numerous bilateral sub-retinal and sub-RPE deposits were seen. (Fig. 1b, e).

**Fig. 2:**
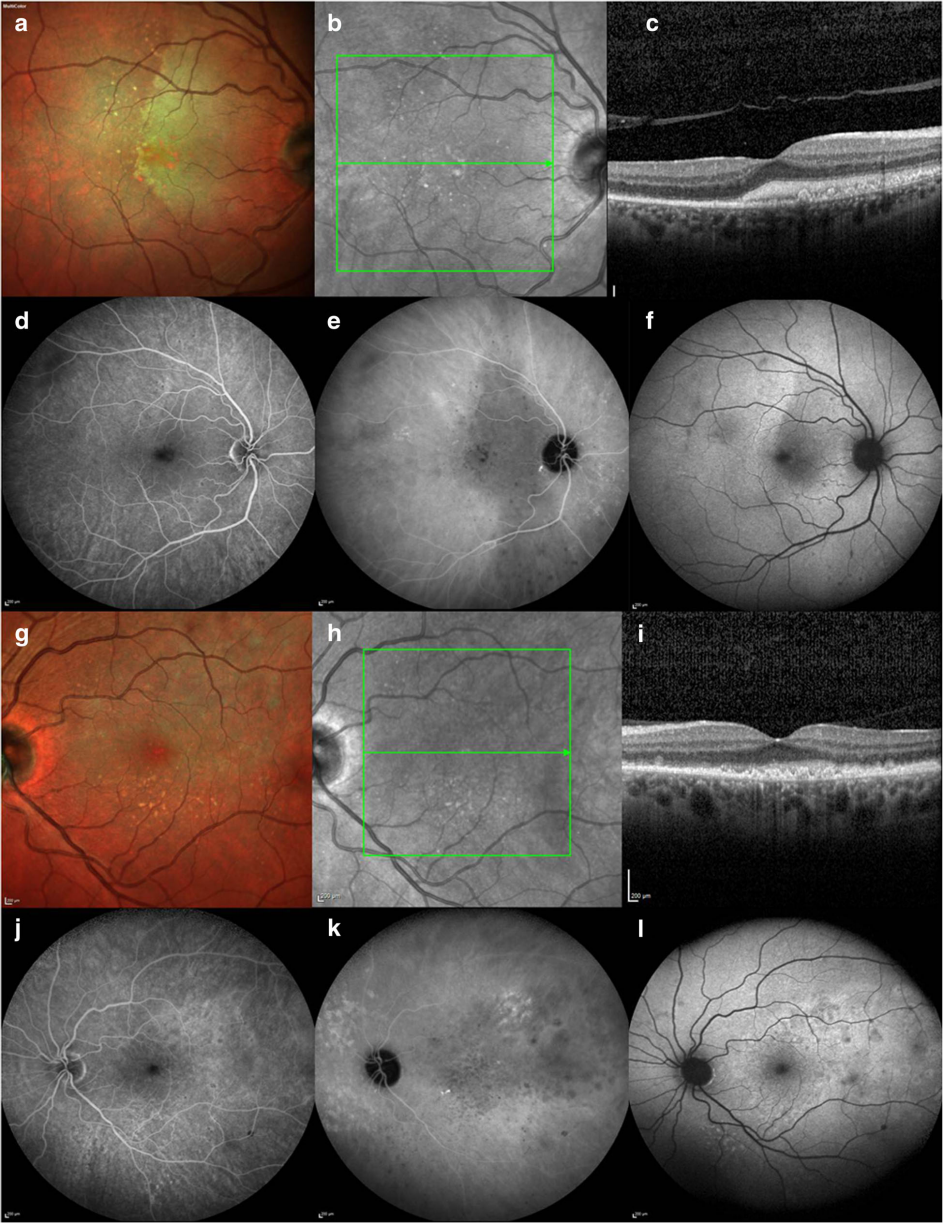
Multi-modal imaging four months after the initial presentation (Spectralis, Heidelberg Engineering Inc., Germany). **a**. MultiColor scanning laser image revealed a large deep, white retinal lesion and multiple yellow retinal deposits. **b**. Red-free image revealed more numerous sub-retinal deposits. **c**. OCT of the right macula lesion demonstrated significant sub-retinal hyper-reflective material and underlying RPE irregularities. **d**. Right eye FFA with hypofluorescent spots corresponding with the yellow retinal deposits. **e**. ICG showing an area of diffuse hypocyanescence corrseponding with the large lesion observed in (2a) was noted with multiple defined areas of hypocyanescence at the sites of observed deposits. **f**. FAF hypoautoflourescence within the large retinal lesion and smaller punctate areas of hypo-autofluorescence. **g** and **h**. Left eye MultiColor and red-free image image with multiple small deep retinal deposits. **i**. Left macula OCT with RPE irregularity and sub-retinal and sub-RPE hyper-reflective changes. Left eye FFA, ICG and FAF demonstrated changes within the areas of OCT abnormality and these were more extensive than those observed on clinical and MultiColour examination: FFA showed multiple hypofluorescent spots (**j**); a diffuse area of macular hypocyanescence and multiple small spots of hypocyanescence were noted on ICG (**k**) and diffuse hyper-autofluorescence corresponding with sub-RPE lesions and focal areas of hypoautofluorescence in areas of sub-retinal lesions were seen on FAF (**l**)

One week later, significant evolution of the right lesion morphology was observed: the temporal margin appeared more defined with re-emergence of a bright linear edge; the lesion had increased in size; new sub-retinal deposits were identified (Fig. 1c) and the lesion appeared to have migrated supero-nasally across the peri-papillary area, maintaining the outline shape temporally of the earlier lesion. Within the previously involved area, the sub-retinal lesion clinically appeared to have resolved and OCT demonstrated resolution of the hyper-reflective change with the RPE intact. (Fig. 1c, 2a-c ) In the left, the lesion inferior to the fovea had evolved and was replaced by multiple small defined sub-retinal and sub-RPE deposits. (Figs. 1f, 2g-i) Fundus fluorescein angiogram (FFA) and indocyanine green angiography (ICG) showed multiple hypofluorescent spots corresponding to the sub-retinal/ sub-RPE deposits. (Fig. 2d-e, j-k) These lesions were more numerous than those clinically detectable. On FAF, there were hypo- and hyperautofluorescent spots corresponding to sub-retinal and sub-RPE deposits respectively. (Fig. 2f, l) The patient was referred to the uveitis service for opinion.

Prior to review, the patient developed acute confusion and impaired neurological function. Magnetic resonance imaging (MRI) of the brain, lumbar puncture and urgent brain biopsy confirmed the diagnosis of primary CNS diffuse large B-cell lymphoma.

**Fig. 3:**
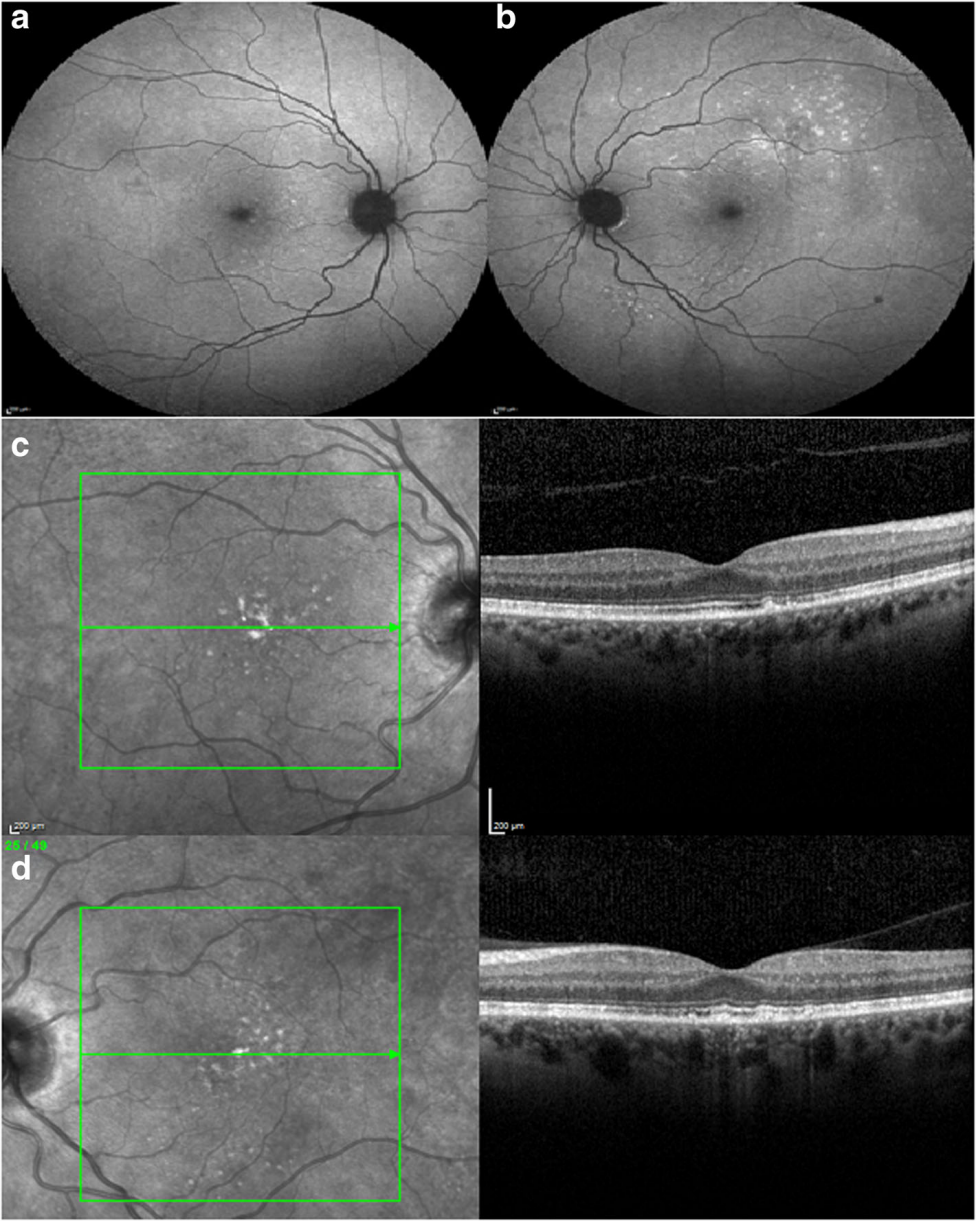
Multi-modal imaging post-treatment. **a** and **b**. Right and left eye FAF imaging with peristent areas of hyper-autofluorescence. **c** and **d**. Right and left red-free imaging with persistent macular lesions and corresponding RPE irregularity and sub-RPE deposits on OCT imaging. OCT demonstrated resoultion of the sub-retinal hyper-reflective changes and restoration of outer retinal structures, including the EZ and external limiting membrane

He underwent MATRix (methotrexate, cytarabine, thiotepa, rituximab) chemotherapy and brain and orbital radiotherapy. During treatment the patient noticed a significant and improvement in his visual symptoms. His last recorded BCVA, at 12 months follow up, was 6/7.5 right and 6/6 left. Clinical examination and repeat imaging demonstrated resolution of the large retinal lesions and reduced number of sub-RPE lesions in the both eyes with complete restoration of the outer retinal architecture on OCT (Fig. 3a to d).

## Discussion

The clinical and imaging findings, including spectral domain OCT, detailed in this case provide an illustrative example of atypical disease presentation and evolution in PVRL. In particular, the observation of both spontaneous resolution of the large subretinal lesion, on clinical and OCT examination, and subsequent lesion migration is also very unusual in PVRL. Furthermore, the retinal lesions had unique and particularly distinct morphological features that evolved over time.

Retinal infiltration in PVRL may involve the intra-retinal, sub-retinal or sub-RPE space [9]. Lymphoma infiltrates are classically creamy, white or orange and may be a single lesion or confluent lesions [9]. The sub-retinal lesion morphological characteristics in our patient were unusual, particularly the appearance of the first sub-retinal lesion, with a sharp geometric-like edge and a bright-white border and re-appearance of this unusual margin in the migrating sub-retinal lesion, which to the best of our knowledge had not been reported in literature. Initially, commotio retina was considered, due to the appearance of retinal whitening and OCT disruption of the outer retina followed by apparent spontaneous lesion regression. Disrupted outer retina on OCT, specifically at the level of the EZ and subsequent EZ restoration has been described in commotio retinae [10]. However, concurrent sub-RPE deposits and subsequent disease behaviour and evolution supported an alternative diagnosis.

Spontaneous regression of tumour in any malignancy is unusual. This phenomenon has been described in a very limited number of cases of intraocular lymphoma [11–13]. The exact mechanism for this phenomenon is unknown but it is hypothesised to be due to tumour control by the host immune system, primarily by the CD8+ T cell and Natural Killer cells [14]. In two reports regression diagnosis was based on histopathological absence of lymphomatous cells; [11, 13] this observation may reflect an immune response but it is recognised that pathological diagnosis can be challenging due to paucity of cells and cellular degeneration and this may account for the authors’ findings. Multimodal imaging findings in such cases is extremely limited [12]. Mantopoulos and Cebulla described photography and OCT evidence of spontaneous regression of a large sub-RPE lesion in PVRL leaving RPE atrophy at the lesion site [12]. As seen in our patient, lesion regression coincided with appearance of new sub-retinal and sub-RPE lesions, indicative of disease evolution. In our patient, clinical resolution of sub-retinal lesion with restoration of the normal retinal appearance and accompanying OCT resolution of hyper-reflective change suggests spontaneous regression. In contrast to the earlier report, the RPE remained intact in our patient, with no atrophy; this may reflect the different infiltration location.

Prior to treatment, we observed an interesting pattern of rapid sub-retinal lesion migration. The lesion shifted in location and the observed edge corresponded with the margin of the hyper-reflective sub-retinal changes on OCT. Following migration, the previously involved retina appeared clinically normal and OCT showed resolution of sub-retinal hyper-reflective change. Following treatment, full resolution of sub-retinal changes was observed on clinical and OCT examination, with no evidence of recurrence. In other reported cases, sub-retinal fibrosis and RPE atrophy have been described following resolution of sub-retinal infiltrate but have not been detected in our case [7].

## Conclusion

We have demonstrated the utility of multi-modal imaging for assessing retinal disease in PVRL including monitoring disease behaviour and response to treatment. OCT imaging and FAF identified more extensive disease than evident on clinical examination, including sub-RPE lesions and disrupted EZ beyond clinically observed lesion margins (Fig. 1a). In conclusion, diagnosis of this condition may be challenging, particularly if spontaneous lesion regression is detected. Lymphoma must be considered in the differential diagnosis of unusual subretinal lesions even in the absence of any vitreous involvement.
